# CysLT_1 _receptor-induced human airway smooth muscle cells proliferation requires ROS generation, EGF receptor transactivation and ERK1/2 phosphorylation

**DOI:** 10.1186/1465-9921-7-42

**Published:** 2006-03-22

**Authors:** Saula Ravasi, Simona Citro, Barbara Viviani, Valérie Capra, G Enrico Rovati

**Affiliations:** 1 Laboratory of Molecular Pharmacology, Section of Eicosanoid Pharmacology, Department of Pharmacological Sciences, University of Milan, Via Balzaretti 9, 20133 Milan, Italy; 2 Laboratory of Toxicology, Department of Pharmacological Sciences, University of Milan, Via Balzaretti 9, 20133 Milan, Italy

## Abstract

**Background:**

Cysteine-containing leukotrienes (cysteinyl-LTs) are pivotal inflammatory mediators that play important roles in the pathophysiology of asthma, allergic rhinitis, and other inflammatory conditions. In particular, cysteinyl-LTs exert a variety of effects with relevance to the aetiology of asthma such as smooth muscle contraction, eosinophil recruitment, increased microvascular permeability, enhanced mucus secretion and decreased mucus transport and, finally, airway smooth muscle cells (ASMC) proliferation. We used human ASMC (HASMC) to identify the signal transduction pathway(s) of the leukotriene D_4 _(LTD_4_)-induced DNA synthesis.

**Methods:**

Proliferation of primary HASMC was measured by [^3^H]thymidine incorporation. Phosphorylation of EGF receptor (EGF-R) and ERK1/2 was assessed with a polyclonal anti-EGF-R or anti-phosphoERKl/2 monoclonal antibody. A Ras pull-down assay kit was used to evaluate Ras activation. The production of reactive oxygen species (ROS) was estimated by measuring dichlorodihydrofluorescein (DCF) oxidation.

**Results:**

We demonstrate that in HASMC LTD_4_-stimulated thymidine incorporation and potentiation of EGF-induced mitogenic signaling mostly depends upon EGF-R transactivation through the stimulation of CysLT_1_-R. Accordingly, we found that LTD_4 _stimulation was able to trigger the increase of Ras-GTP and, in turn, to activate ERK1/2. We show here that EGF-R transactivation was sensitive to pertussis toxin (PTX) and phosphoinositide 3-kinase (PI3K) inhibitors and that it occurred independently from Src activity, despite the observation of a strong impairment of LTD_4_-induced DNA synthesis following Src inhibition. More interestingly, CysLT_1_-R stimulation increased the production of ROS and *N*-acetylcysteine (NAC) abolished LTD_4_-induced EGF-R phosphorylation and thymidine incorporation.

**Conclusion:**

Collectively, our data demonstrate that in HASMC LTD_4 _stimulation of a G_i/o _coupled CysLT_1_-R triggers the transactivation of the EGF-R through the intervention of PI3K and ROS. While PI3K and ROS involvement is an early event, the activation of Src occurs downstream of EGF-R activation and is followed by the classical Ras-ERK1/2 signaling pathway to control G1 progression and cell proliferation.

## Background

Cysteine-containing leukotrienes (cysteinyl-LTs), i.e. LTC_4_, LTD_4 _and LTE_4_, are pivotal inflammatory mediators formed through the 5-lipoxygenase pathway of arachidonic acid and contribute to the pathogenesis of asthma [1]. In particular, cysteinyl-LTs are very potent constrictors of human bronchi not only *in vitro*, but also *in vivo*, both in normal and in asthmatic individuals [2]. Recently, the focus in asthma therapy shifted from the short-term relief of acute bronchoconstriction to the long-term management of chronic inflammation [3]. Hallmark of this process is the infiltration of inflammatory cells, predominantly eosinophils, mast cells, and lymphocytes [4], but also a significant airway remodeling [5,6]. The features of airway remodeling include subepithelial fibrosis, elevated numbers and volume of mucous cells in the epithelium, increased amounts of airway smooth muscle cells (ASMC), and increased vascularization of the airway wall [7-9]. Another peculiar characteristic of chronic asthma is known to be the airway hyperresponsiveness (AHR) [10], whose underlying mechanism certainly involves the hypersensitivity to G protein coupled receptors (GPCRs) contractile agonists such as carbachol, histamine, acetylcholine and cysteinyl-LTs [11].

Furthermore, numerous contractile agents have been shown to induce proliferation of ASMC in culture [12], suggesting that a persistent stimulation with contractile agonists and inflammatory agents might play an important role in triggering and sustaining airway remodeling that, in turn, contribute to AHR in asthma [3,13]. In particular, it has been suggested that LTD_4 _is able to augment growth factor-induced human ASMC (HASMC) proliferation through an "atypical" CysLT-R [14] or to directly induce proliferation in cytokine primed HASMC through a classical CysLT_1_-R [15]. Moreover, very recently montelukast, a potent CysLT_1_-R antagonist clinically used in the therapy of asthma [16,17], has been demonstrated to inhibit allergen-induced airway remodeling in an *in vivo *mouse model of asthma [18]. However, the precise molecular basis for LTD_4_-induced HASMC growth is not known, while the mechanism underlying other spasmogens seems to vary from agonist to agonist in addition to cell type and might involve many different and sometimes parallel pathways [5,19].

It is known that LTD_4 _acts through two specific GPCRs, namely CysLT_1 _and CysLT_2 _[20,21], which appears to be mainly coupled to G_q/11 _and thus to intracellular Ca^2+ ^elevation in recombinant systems, but also to G_i/o _in some natural expressing systems [22,23]. This clearly reveals heterogeneity/promiscuity of coupling for this class of receptors as already demonstrated for many other GPCRs, in particular when comparing natural and recombinant systems [24].

A number of studies have advanced the concept that GPCRs are mediators of cell growth by demonstrating their potential to activate MAPKs, particularly the ERK1/2 [25]. Indeed, recently, we have demonstrated that CysLT_1_-R is able to phosphorylate ERK1/2 and activate Ras through a pertussis toxin (PTX) sensitive G protein in the human monocyte/macrophage-like U937 cells [26]. A number of different mitogenic pathways might link GPCRs to the nucleus, some of them requiring the activation of the small GTP-binding protein Ras or, for G_q _coupled receptors, protein kinase C (PKC) to directly target Raf-1 [25]. In some systems, the capacity of GPCRs to transduce a mitogenic response requires a growth factor receptor, such as the epidermal growth factor receptor (EGF-R) and its tyrosine kinase activity [27]. Indeed, it has already been suggested that LTD_4 _is able to transactivate PDGF receptor in mesangial cells, but this activation was somehow attributed to the activation of a CysLT_2_-R [28].

Because of their role in chronic asthma, ASMC represent a clinically relevant cell type in which to examine the effects of LTD_4_-induced activation of cell growth [12]. Both MAPKs and phosphatidylinositol-3-OH-kinase (PI3K) have been shown to mediate mitogen-induced proliferation in these cells [29]. In addition, proliferation synergy by receptor tyrosine kinase (RTK) and GPCR activation by numerous inflammatory or contractile agents have been demonstrated in HASMC, although it did not seem to be correlated to EGF-R transactivation [30-32].

In this report we investigated the role and the mechanism(s) by which LTD_4 _induces HASMC proliferation. We show that this lipid mediator is able to induce HASMC proliferation by itself activating a CysLT_1_-R and that the mitogenic effect is dependent upon EGF-R phosphorylation. We also demonstrated that LTD_4 _induces reactive oxygen species (ROS) formation. Finally, we show here that, despite the possible contribution of G_q/11_-mediated pathway, both transactivation and DNA synthesis are predominantly mediated by a G_i/o _protein and that CysLT_1_-R-induced activation involves, downstream of EGF-R, the classical Src-Ras-ERK signaling pathway.

## Methods

### Materials

Cell culture supplies, EGF, *N*-acetylcysteine (NAC), CRM197 and anti-smooth muscle α-actin antibody were purchased from Sigma Chemical Co (St. Louis, MO); anti EGF-R, p-EGF-R (Tyr 1173) and ERK1/2 were from Santa Cruz Biotechnology (Santa Cruz, CA). Ras activation assay kit containing a GST fusion protein corresponding to the human RBD of Raf1 and a pan-Ras mouse monoclonal antibody (clone RAS10) was purchased from Upstate biotechnology (Lake Placid, NY); Anti-p-ERK1/2 (Thr 202 and Tyr 204) monoclonal antibody is from Cell Signaling Technology (Beverly, MA); Fetal bovine serum (FBS), TriZol^® ^Reagent, CysLT_1 _receptor oligonucleotide primers and Platinum Taq DNA Polymerase were from Life Technologies (NY, USA); LTD_4 _from Cayman Chemical Co. (Ann Arbor, MI); zafirlukast, pranlukast and montelukast were a gift from Merck & Co. (West Point, PA). AG1478, pertussis toxin, PD98059, U73122 and genistein, were from Calbiochem (La Jolla, CA); PP1 from Biomol (Plymouth Meeting, PA). DCFH-DA (6-carboxy-2',7'-dichloro-dihydrofluorescein diacetate, di(acetoxymethyl ester)) was from Molecular Probes (Eugene, OR). Ultima Gold scintillation liquid and [^3^H] ICI198,615 were from Perkin Elmer life sciences (Boston, MA); MMLV-Reverse Transcriptase RETROscript™ for RT-PCR was from Ambion (Austin, TX); the protease inhibitor complex Complete™ from Roche Applied Sciences (Basel, Switzerland). Reagents and films for chemiluminescence and [^3^H]Thymidine were from Amersham Bioscience (Piscataway, NJ). All reagents and supplies for electrophoresis and DC™ Protein assay were from Bio-Rad Laboratories (Richmond, CA).

### Cell culture

Smooth muscle cells from human bronchi were purchased from Cambrex (Walkersville, MD) or isolated in our laboratory as previously described [33]. Briefly, macroscopically normal lung fragments were obtained at thoracotomy. Third order bronchi were removed under sterile conditions, the connective tissue and the epithelium were removed and the smooth muscle cut into pieces approximately 10 mg each. The explants were grown at 37°C in a humidified atmosphere of 5% CO_2 _in Medium 199, additioned with 20% (v/v) FBS, 100 U/ml penicillin and 100 μg/ml streptomycin in 25 cm^2 ^culture flask. The primary isolates were positively stained with an anti-smooth muscle α-actin antibody to assess the identity of the cultures. Thereafter, cells were routinely grown in monolayers in MEM supplemented with 10% FBS, 100 U/ml penicillin and 100 μg/ml streptomycin, passaged at a 1:3 ratio in 75 cm^2 ^culture flask and used between the 3^rd ^and 8^th ^passage (our isolates) or between the 3^rd ^and the 10^th ^passage (purchased cells).

### RT-PCR of CysLT_1 _receptors

Total RNA was extracted from HASMC using "TRIZOL^® ^Reagent", according to the manufacturer's instructions. After denaturation (75°C, 3 minutes), 1–2 μg of total purified RNA was retrotranscribed in the presence of MMLV-Reverse Transcriptase (5 U/μl) in optimized reaction conditions (RT-Buffer: 50 mM Tris-HC1, pH 8.3, 75 mM KC1, 3 mM MgCl_2_, 5 mM DTT, 2 mM dNTPs, 0.5 U/μl RNAse inhibitors 42–44°C, 1 h). Specific amino- and carboxyl-terminal primers for hCysLT_1 _receptor (N-terminal, 5'-ATGGATGAAACAGGAAATCTGACAG-3'; C-terminal, 3'-CTATACTTTACATATTTCTTCTCC-5') and for CysLT_2 _receptor (N-terminal, 5'-ACCTTCAGCAATAACAACAGC-3'; C-terminal, 3'-CTTTATGCAGTCTGTCTTTGC-5') have been selected on the basis of the sequences previously published [34,35]. The PCR mediated amplification of cDNA employed Taq Platinum Polymerase (0.03 U/μl), in optimized conditions (PCR-Buffer: 20 mM Tris-HCl, pH 8.4, 75 mM KC1,0.2 mM dNTPs, 2 mM MgCl_2_, 0.2 μM forward and reverse primers) using a Bio-Rad/I-Cycler PCR system. Specific cDNA fragments of 948 bp and 1117 bp were amplified (25–30 cycles: *denaturation: *95°C, 30 sec; *annealing *60°C, 20 sec; *extension*: 68°C, 45 sec) and visualized after electrophoresis in 1% agarose gels by UV irradiation.

### Binding studies

Radioligand binding was performed at equilibrium on membranes of HASMC (0.25 mg/samples) prepared as previously described [36], in a final volume of 500 μl of PBS containing 2.5 mM CaCl_2_, 2.5 mM MgCl_2_, 10 mM glycine and 20 mM penicillamine. Equilibrium binding studies were performed at 25°C for 1 h with a mixed type binding protocol, obtained by combining both saturation (0.1–1 nM of [^3^H]ICI198,615) and competition (3 nM – 1 μM of ICI198,615) protocols in a single curve [37]. Unbound ligand was separated by rapid vacuum filtration using a Brandel cell harvester and radioactivity measured by liquid scintillation counting. Analysis of binding data was performed by means of the computer program LIGAND (see Statistical analysis). The protein content was determined by Lowry quantization assay. Non specific binding ranged between 40 and 50% of the total binding.

### [^3^H] Thymidine incorporation assay

[^3^H]Thymidine incorporation assay was performed as previously described [31]. Briefly, cells were subcultured into 24 well plates and subconfluent cells were serum-starved for 48 hours to synchronize the entire population cell cycle. Medium was then replaced with MEM containing 1% FBS and cells stimulated with LTD_4 _(two pulses at 4 hrs interval) and EGF (single pulse) at the indicated concentration for 48 hours and incubated with [^3^H]thymidine for the last 4 hours at a final concentration of 1 *μ*Ci ml^-1^. Cells were then washed twice with ice-cold phosphate buffered saline to rinse loosely associated radioactive tracer. Acid-soluble radioactivity was removed by 20 minutes treatment with 5% trichloroacetic acid at 4°C followed by a two-step washing with 95% ethanol. The acid-insoluble portion was recovered by 60 minutes digestion with 2% Na_2_CO_3 _in 0.1 M NaOH. The radioactivity was then measured by liquid scintillation counting.

### Phosphorylation of EGF-R

Phosphorylation of EGF-R was performed as previously described [31]. Briefly, subconfluent cells in 60-mm dishes were serum-starved for 48 h, and 2 h before stimulation the medium was replaced by MEM containing 0.1% fetal bovine serum. The cells were then stimulated at 37°C with LTD_4 _or EGF at the concentrations indicated for 5 min. AG1478 was preincubated for 1 hour before the addition of EGF. Monolayers were then placed on ice, washed twice with phosphate buffered saline, lysed (20 mM TrisHCl pH 7.5, 1 mM dithiotreitol, 2 mM EGTA, 20 mM EDTA, 1 mM phenylmethylsulfonyl fluoride, 1 mM Na_3_VO_4 _and the protease inhibitor complex Complete™) and sonicated four times for 15 s. The samples were subsequently diluted in Laemmli buffer, resolved by SDS-PAGE (sodium dodecyl sulfate polyacrilamide gel electrophoresis, 20 *μ*g/lane – 6% gel) and transferred to a nitrocellulose membrane. Immunoblotting was performed with a polyclonal anti-EGF-R antibody at a concentration of 0.2 *μ*g/ml for 18 h at 4°C and immune complex was detected by chemiluminescence using horseradish peroxidase-conjugated goat anti-rabbit IgG (immunoglobulin G) as a secondary antibody. The membrane was stripped with stripping buffer and re-blotted with polyclonal anti-EGF-R antibody.

### ERK1/2 phosphorylation

ERK1/2 phosphorylation was performed as previously described [32]. Briefly, confluent cells into 35 mm dishes were serum-starved for 48 h, preincubated with the inhibitors for the indicated time and stimulations at 37°C were terminated by addition of ice-cold lysis buffer (see above). Thereafter, the lysates were sonicated four times on ice for 15 sec, the protein content was measured and compensated for prior to SDS-PAGE. Cell lysates were solubilized by boiling at 95°C for 5 min in Laemmli buffer and subjected to electrophoresis on 15% polyacrylamide gel. The separated proteins were transferred to a nitrocellulose membrane. Membranes were then blocked for 1 hour with 5% non-fat dried milk at room temperature and then incubated overnight at 4°C with an anti-ERK2 or anti-phosphoERK1/2 monoclonal antibody at the concentration of 1 ng/ml. Next, membranes were washed extensively and incubated with horseradish peroxidase-conjugated goat anti-mouse IgG as a secondary antibody for 1 hour at room temperature. After three washes, the immunoreactive proteins were visualized by chemiluminescence.

### Ras activation

Ras activation assay was performed following the affinity precipitation protocol provided by the manufacturer (Ras pull down assay kit) as previously described [26]. Briefly, cells were serum-starved overnight, treated with appropriate stimuli, and then lysed as previously described (see above). Lysates (1 mg/ml of total cell proteins in each sample) were incubated with 10 μg of Raf-1 RBD for 45 minutes at 4°C and then centrifuged for 15 seconds at 14000 × *g *to pellet the agarose beads. After discarding the supernatant, agarose beads were washed with 1 ml PBS and then the pellets were resuspended in 2X Laemmli sample buffer containing DTT, boiled for 5 minutes, and finally centrifuged for 15 sec at 14000 × *g*. The supernatant was collected and cellular proteins resolved by SDS-PAGE using 11% (w/v) acrylamide and analyzed by western blotting (see above).

### ROS measurement in living cells

HASMC were loaded with 10 μM DCFH-DA in saline buffer supplemented with 0.1% of bovine serum albumin for 1 hat 37°C (stained). At the end of the incubation, cells were washed in PBS and oxidative activity was assessed as follows. The production of reactive oxygen species (ROS) was measured by the intensity of DCF emission at 525 nm (excitation 503 nm – Perkin-Elmer LS 50B) in both stained and unstained cells. Results are expressed as the difference in fluorescence (in arbitrary units, AU) calculated as AU = [I_t5 _- I_t0_], where I represents the intensity of fluorescence at the specified time points.

### Statistical analysis and experimental design

Ligand binding studies were analyzed using LIGAND computer program. Non specific binding was calculated by LIGAND as one of the unknown parameters of the model. Selection of the best fitting model and evaluation of the statistical significance of the parameter difference was based on the F-test for the extra sum of square principle. Parameter errors are always expressed in % coefficient of variation (%CV). The curve shown was computer generated. Statistical comparison of two groups was performed using an independent t test; multiple groups were analyzed using one way ANOVA followed by either Bonferroni or Dunnett post hoc test. Data are expressed as means ± S.E.M. Each experiment was performed at least three times in triplicate (were possible) on at least two different cell lines. Basal condition refers to cells unexposed to antagonists or inhibitors and, where necessary, vehicle-treated.

## Results

### LTD_4_-induced increase in DNA synthesis

**Figure 1:**
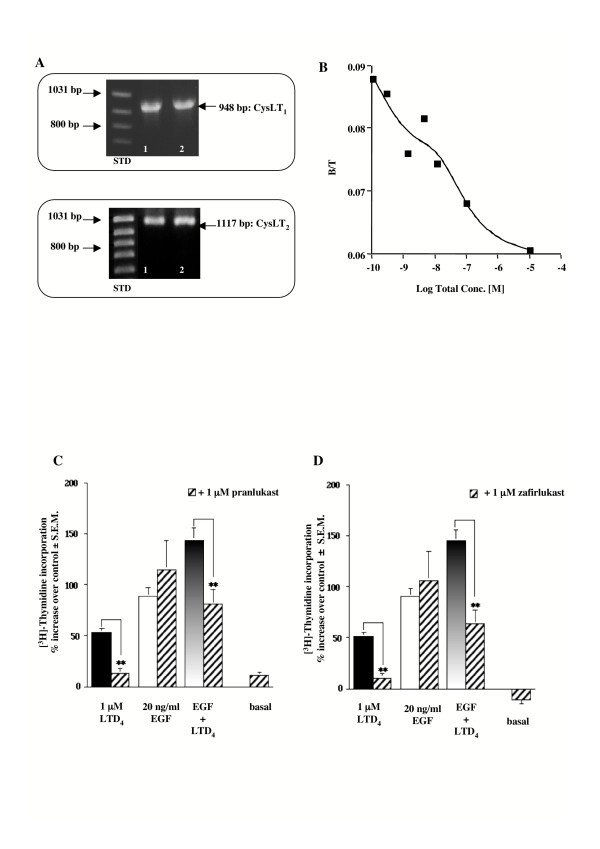
**RT-PCR, [^3^H]ICI198,615 binding and LTD_4_-induced [^3^H]thymidine incorporation**. (A) Final RT-PCR products obtained using inner primers for human CysLT_1_-R (Upper Panel) and CysLT_2_-R (Lower Panel). The PCR mediated amplification of cDNA produced the expected 948 bp (CysLT_1_) and 1117 bp fragments (CysLT_2_), which were visualized upon agarose gel, by UV irradiation. Lane 1, purchased HASMC and lane 2, HASMC isolated in our lab; STD, standard. (B) Equilibrium binding curve of [^3^H]ICI198,615 in membranes from HASMC. Mixed type binding curves were performed using 0.1–1 nM [^3^H]ICI198,615 (saturation part of the curve) and 1 nM – 1 μM of unlabeled ICI198.615 (competition part of the curve). Binding is expressed as the ratio of bound ligand concentration over total ligand concentration, (B/T, dimensionless), vs. the logarithm of total ligand concentration. B (in M) is the sum of "hot", "cold", and non-specific binding; T (in M) is the sum of "hot" and "cold" ligand incubated. Data shown are representative of two independent experiments simultaneously analyzed with LIGAND. (C-D) Effect of LTD_4_, EGF, and CysLT_1_-R antagonists on [^3^H]thymidine incorporation in HASMC. Increase of [^3^H]thymidine incorporation induced by 1 μM LTD_4 _and 20 ng/ml EGF alone or in combination, in the absence and presence of 1 μM of the antagonists pranlukast (C) and zafirlukast (D) (30 min pretreatment). Control is represented by MEM additioned with 1% FBS. The results are presented as mean ± S.E.M. of at least three experiments performed in triplicate on two different cell lines. **P < 0.01 (one-way ANOVA).

In order to confirm whether the different HASMC utilized expressed an LTD_4 _receptor, we routinely performed a RT-PCR reaction to amplify the human CysLT_1_-R specific DNA sequence. Fig. 1A (Upper Panel) shows the expected product of 948 bp. Indeed, HASMC also express the CysLT_2_-R (Fig. 1A, Lower Panel). We performed equilibrium binding studies in membranes from HASMC using [^3^H]ICI198,615 as labeled ligand (Fig. 1B). Computer analysis of the mixed type curve revealed the presence of two classes of binding sites, as previously reported [38]. The high affinity binding site, representative of the CysLT_1_-R [20], exhibited a K_d _= 0.16 nM ± 68 %CV and a B_max _= 6.6 fmol/mg/prot ± 82 %CV. Both parameters were as expected for a constitutive CysLT_1_-R.

In HASMC 1 μM LTD_4 _was able to produce an increase in [^3^H]thymidine incorporation (53% ± 4.1 S.E.M. increase vs. control; n = 27), and to potentiate EGF-induced proliferation (39% ± 4.7 S.E.M. increase vs. EGF alone; n = 15). Two pulses of 1 μM LTD_4 _has been utilized because it is known that LTD_4 _is highly unstable and rapidly metabolized to LTE_4_, a much weaker partial agonist at the CysLT_1_-R. Furthermore, 30 minutes pretreatment with 1 μM of the two CysLT_1_-R antagonists pranlukast and zafirlukast strongly prevented LTD_4_-induced HASMC [^3^H]thymidine incorporation (86% ± 15 S.E.M. and 75% ± 12, respectively), as well as its potentiating effect of the EGF-induced DNA synthesis (Fig. 1C–D), demonstrating that LTD_4 _is mostly acting through a classical CysLT_1_-R. As expected, neither pranlukast nor zafirlukast were able to influence the mitogenic effect of EGF.

### LTD_4_-mediated EGF-R phosphorylation

**Figure 2:**
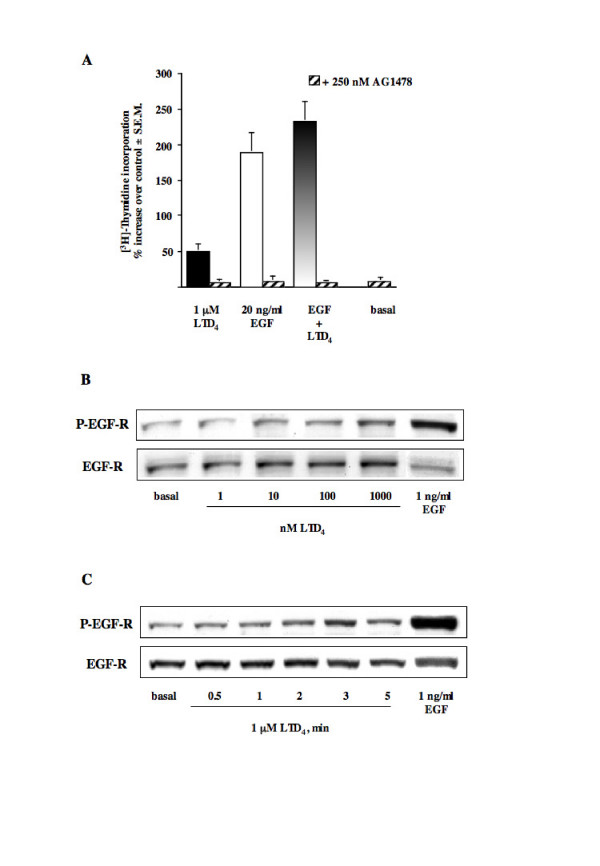
**LTD_4_-induced EGF-R phosphorylation**. (A) Increase of [^3^H]thymidine incorporation induced by 1 μM LTD_4 _and 20 ng/ml EGF alone and in combination, in the absence and presence of 250 nM AG1478 (1 h pretreatment). Control is represented by MEM additioned with 1% FBS. The results are presented as mean ± S.E.M. of three experiments performed in triplicate on two different cell lines. (B) Concentration-response curve of EGF-R phosphorylation induced by 3 minutes treatment with the indicated concentrations of LTD_4_. (C) Time-course of EGF-R phosphorylation induced by 1 μM LTD_4_. For B and C 1 ng/ml EGF was used as an internal control and the experiments were repeated twice.

To investigate a possible transactivation of the EGF-R by LTD_4 _in HASMC, we tested the EGF-R tyrosine kinase inhibitor AG1478. Figure 2A shows that 1 hour pretreatment with 250 nM AG1478 fully inhibited DNA synthesis induced by LTD_4 _and EGF alone or by their combination.

**Figure 3:**
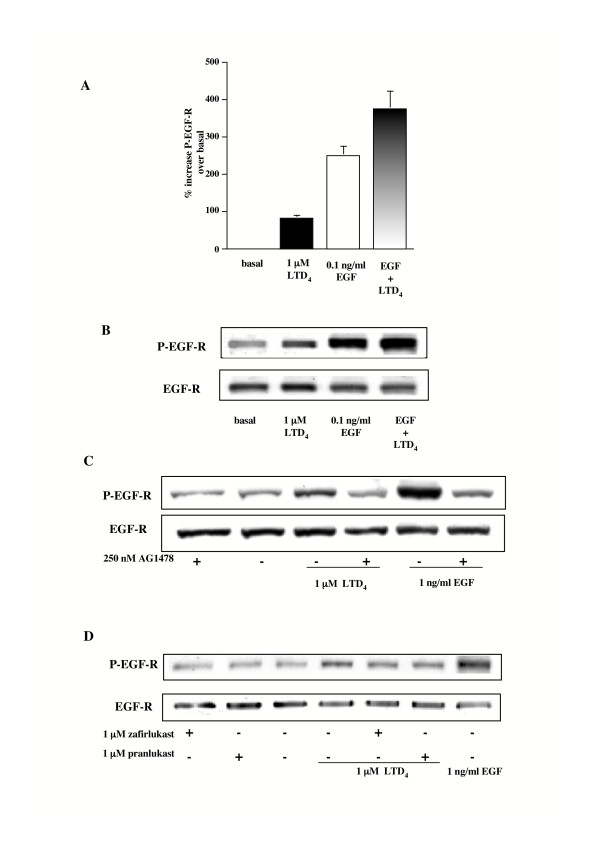
**Effect of AG1478 and CysLT_1_-R antagonists on LTD_4_-induced EGF-R phosphorylation**. (A) EGF-R phosphorylation induced by 1 μM LTD_4 _(n = 20) and 0.1 ng/ml EGF (3 minutes) alone and in combination (n = 4). The results are presented as mean ± S.E.M. (B) Representative experiment. (C) EGF-R phosphorylation induced by 1 μM LTD_4_, 1 ng/ml EGF (3 minutes), in the absence and presence of 250 nM AG1478 (1 h preincubation). D) EGF-R phosphorylation induced by 1 μM LTD_4 _in the absence and presence of 1 μM of the antagonists zafirlukast and pranlukast (30 minutes pretreatment). 1 ng/ml EGF was used as an internal control. (C-D) The results presented are representative of at least three experiments performed on different cell lines.

Because we were interested in investigating whether the EGF-R can function as a downstream signaling partner of cysteinyl-LTs, therefore acting as a point of convergence for heterogeneous signaling pathways, we used immunoblot analysis to assess the phosphorylation state of this receptor following the treatment of the cells with LTD_4_. As shown in Figure 2B and 2C, EGF-R tyrosine phosphorylation was concentration- and time-dependent, starting as early as 2 min and peaking at 3 min at a concentration of 1 μM. Thus, all subsequent experiments were performed stimulating cells with 1 μM LTD_4 _for 3 min, which produced an average EGF-R phosphorylation of 80% ± 7.8 S.E.M., (Fig. 3A, n = 20). Furthermore (Fig. 3B), LTD_4 _also induced potentiation of EGF-stimulated autophosphorylation (47% ± 4.8 S.E.M. increase vs. 0.1 ng/ml EGF, Fig. 3A, n = 4), a result in agreement with LTD_4 _potentiation of EGF-stimulated DNA synthesis. Unsurprisingly, 1 hour pretreatment with 250 nM AG1478 totally inhibited LTD_4_-induced EGF-R phosphorylation (Fig. 3C). In addition, LTD_4 _effect was significantly prevented by 1 μM zafirlukast and pranlukast (~70% and ~60%, respectively) (Fig. 3D), a result again in good agreement with the CysLT_1_-R antagonist inhibition of thymidine incorporation (see above).

### LTD_4_-induced ERK1/2 phosphorylation

**Figure 4:**
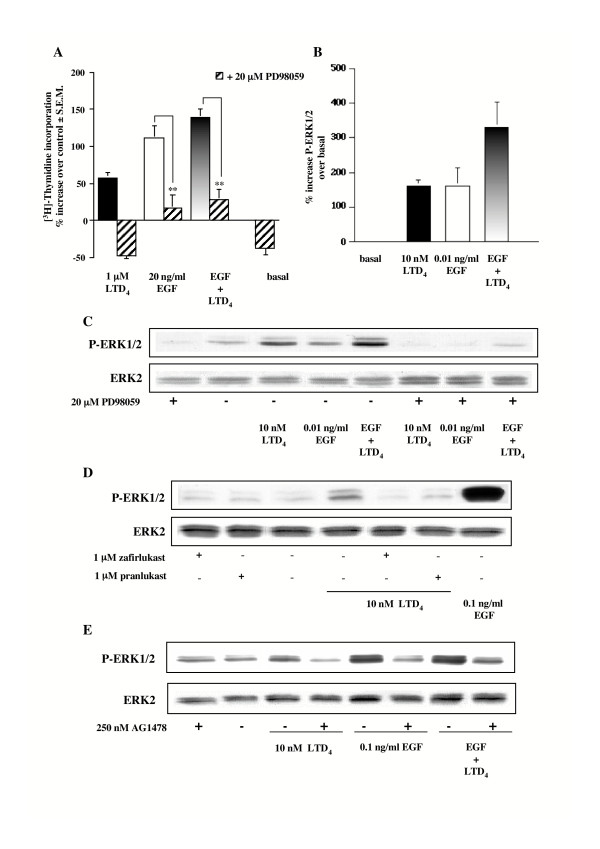
**LTD_4_-induced ERK1/2 phosphorylation**. (A) Increase of [^3^H]thymidine incorporation induced by 1 μM LTD_4 _and 20 ng/ml EGF alone and in combination in the absence and presence of 20 μM PD98059 (1 h preincubation). Control is represented by MEM additioned with 1% FBS. The results are presented as mean ± S.E.M. of three experiments performed in triplicate on two different cell lines. **P < 0.01 (one-way ANOVA). (B) ERK1/2 phosphorylation induced by 10 nM LTD_4 _(n = 12) and 0.01 ng/ml EGF (5 minutes) alone and in combination (n = 4). The results are presented as mean ± S.E.M. (C) ERK1/2 phosphorylation induced by 10 nM LTD_4 _and 0.01 ng/ml EGF (5 minutes) alone and in combination in the absence and presence of 20 μM PD98059 (1 h preincubation). (D) Effect of 1 μM zafirlukast and pranlukast (30 minutes pretreatment) on ERK1/2 phosphorylation induced by 10 nM LTD_4 _(5 min). 0.1 ng/ml EGF was used as an internal control. (E) ERK1/2 phosphorylation induced by 10 nM LTD_4_, 0.1 ng/ml EGF (5 minutes), alone or in combination, in the absence and presence of 250 nM AG1478 (1 h preincubation). (D-E) The results presented are representative of at least three experiments performed on two different cell lines.

Because we and others have already suggested that LTD_4 _is able to activate ERK [26,39,40], we tested the effect of a specific MAPK/ERK kinase (MEK1) inhibitor, i.e. PD98059, on the LTD_4_-induced [^3^H]thymidine incorporation. Indeed, 1 hour pretreatment with 20 μM PD98059 fully prevented LTD_4_-induced DNA synthesis (Fig. 4A), as well as the basal level of [^3^H]thymidine incorporation in the presence of 1% FBS. At variance, PD98059 only partially inhibited the effect caused by EGF and by the combined effect of LTD_4 _and EGF.

To confirm the involvement of ERK 1/2, we measured the amount of their phosphorylated form by western blotting. Stimulation of HASMC with10 nM LTD_4 _for 5 minutes (time course and concentration-response curves not shown) produced a maximal ERK1/2 phosphorylation of 162% ± 16 S.E.M. (Fig. 4B n = 12). This effect was completely inhibited by 1 μM zafirlukast and pranlukast (Fig. 4D). Furthermore (Fig. 4C), LTD_4 _also induced potentiation of EGF-stimulated MAPK activation (104% ± 28 increase vs. 0.01 ng/ml EGF; Fig. 4B n = 4).

To test the hypothesis that MAPK activation could be dependent upon EGF-R transactivation, we also tested the effect of AG1478 on LTD_4_-induced ERK1/2 phosphorylation and found that 1 hour pretreatment with 250 nM of the EGF-R phosphorylation inhibitor totally prevented ERK1/2 activation by EGF and LTD_4 _alone or by their combination (Fig. 4E).

The time frame of EGF-R activation (3 min) is fully compatible with that of MAPK activation (5 min). Despite there is disagreement in the literature on the necessity for prolonged activation of the MAPK cascade to produce a significant mitogenic effect [41,42], we recently demonstrated that a rapid and transient activation of ERK following TP receptor stimulation translocates ERK into the nucleus as early as 2 min, where it accumulates in its active form [32]. Thus, these data suggest that that stimulation of the CysLT_1_-R in HASMCs might be important for the control of transcription factors and cell cycle re-entry, especially with other mitogenic stimuli (e.g., EGF), if efficient cell proliferation is to be achieved.

### Effect of PTX on LTD_4_-induced DNA synthesis, EGF-R phosphorylation and ERK1/2 activation

**Figure 5:**
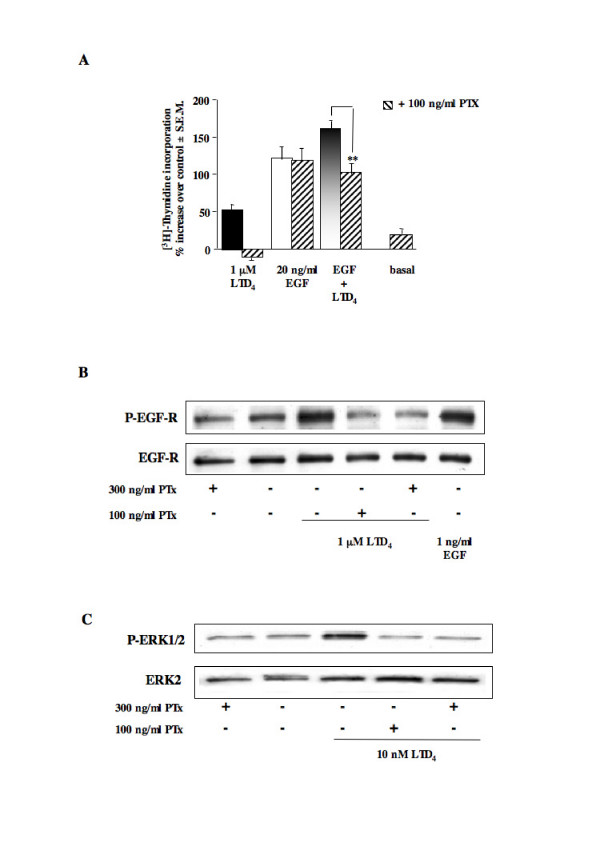
**Effect of PTX on [^3^H]thymidine incorporation, EGF-R and ERK1/2 phosphorylation induced by LTD_4 _or EGF**. (A) Increase of [^3^H]thymidine incorporation induced by 1 μM LTD_4 _and 20 ng/ml EGF alone and in combination in the absence and presence of 100 ng/ml PTX (20 hours pretreatment). Control is represented by MEM additioned with 1% FBS. The results are presented as mean ± S.E.M. of three experiments performed in triplicate on two different cell lines. **P < 0.01 (one-way ANOVA). (B) EGF-R phosphorylation induced by 1 μM LTD_4 _(3 minutes), in the absence and presence of 100 or 300 ng/ml PTX (20 hours pretreatment). 1 ng/ml EGF was used as an internal control. (C) ERK1/2 phosphorylation induced by 10 nM LTD_4 _(5 minutes) in the absence and presence of 100 or 300 ng/ml PTX (20 hours pretreatment). The results presented are representative of at least three experiments performed on two different cell lines.

To identify which class of G protein is involved in LTD_4_-induced [^3^H]thymidine incorporation, we pretreated HASMC with 100 ng/ml PTX for 20 hours. Preincubation of HASMC with PTX fully abolished LTD_4_-induced DNA synthesis (Fig. 5A). On the contrary, the toxin failed to affect EGF mitogenic response, while it reverted [^3^H]thymidine uptake level to the one induced by EGF alone. Furthermore, PTX (either 100 or 300 ng/ml) also fully inhibited LTD_4_-induced EGF-R and ERK1/2 phosphorylation, demonstrating that not only EGF-R transactivation, but also ERK1/2 phosphorylation is totally dependent upon a G_i/o _protein activation (Fig. 5B and 5C, respectively).

### Effect of genistein and PP1 on LTD_4_-induced DNA synthesis, EGF-R and ERK1/2 phosphorylation, and LTD_4_-induced Ras activation

**Figure 6:**
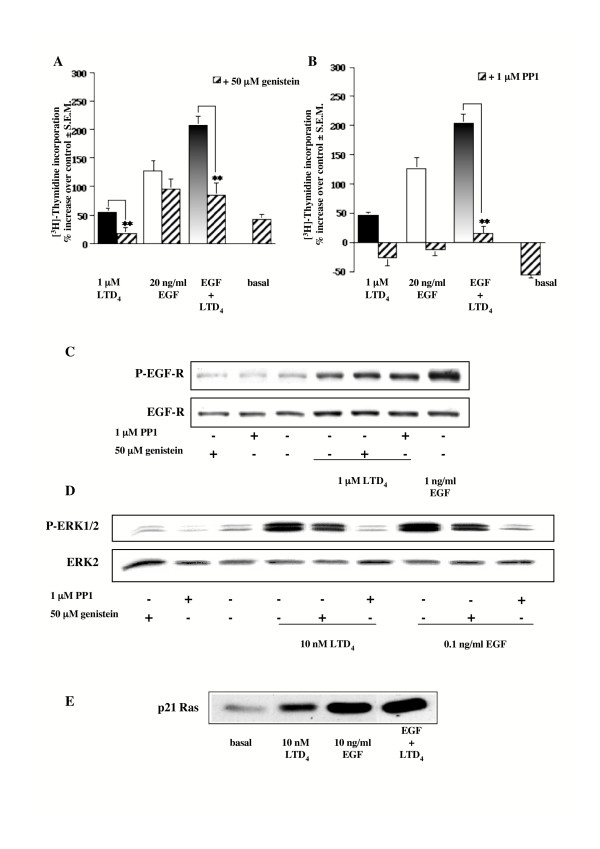
**Effect of genistein and PP1 on [^3^H]thymidine incorporation, EGF-R and ERK1/2 phosphorylation, and Ras activation induced by LTD_4 _or EGF**. (A-B) Increase of [^3^H]thymidine incorporation induced by 1 μM LTD_4 _and 20 ng/ml EGF alone and in combination in the absence and presence of (A) 50 μM genistein or (B) 1 μM PP1 (30 minutes pretreatment). Control is represented by MEM additioned with 1% FBS. The results are presented as mean ± S.E.M. of at least three experiments performed in triplicate on at least two different cell lines. **P < 0.01 (one-way ANOVA). (C) EGF-R phosphorylation induced by 1 μM LTD_4 _(3 minutes), in the absence and presence of 50 μM genistein or 1 μM PP1 (30 minutes pretreatment). 1 ng/ml EGF was used as an internal control. (D) ERK1/2 phosphorylation induced by 10 nM LTD_4 _and 0.1 ng/ml EGF (5 minutes) in the absence and presence of 50 μM genistein or 1 μM PP1 (30 minutes pretreatment). E) Increase of Ras-GTP levels induced by 10 nM LTD_4 _and 10 ng/ml EGF alone and in combination (5 minutes). Activated Ras (p21 Ras-GTP) was co-immunoprecipitated and detected by immunoblotting the same amount of proteins for each sample with a pan-Ras antibody. The results presented are representative of at least three experiments performed on two different cell lines.

It is known that GPCRs might trigger activation of a protein tyrosine kinase (PTK) such as Src in order to activate MAPKs. Thus, we tested the effect of genistein, a broad spectrum PTK inhibitor, and of PP1, a specific Src kinase inhibitor, on LTD_4_-stimulated [^3^H]thymidine incorporation in HASMC. It is clear from Figure 6A and 6B that 30 minutes pretreatment with 50 μM genistein or 1 μM PP1 strongly inhibited DNA synthesis caused by LTD_4 _(> 90%), or by costimulation of EGF and LTD_4 _(> 70%). Furthermore, PP1 and to a lesser extent genistein were able to inhibit the effect induced by EGF alone.

It is also known that a possible pathway leading to EGF-R transactivation by G_i _coupled receptors requires Src kinase activity. However, Figure 6C shows that neither genistein nor PP1 significantly affected LTD_4_-stimulated EGF-R transactivation as well as EGF-induced autophosphorylation (data not shown). At variance, both compounds inhibited LTD_4_- (~33% and > 90%, respectively) or EGF-stimulated (~34% and ~86%, respectively) ERK1/2 phosphorylation in HASMC in a similar way (Fig. 6D).

Finally, through a Ras pull-down assay we directly demonstrated that 10 nM LTD_4 _was able to increase the amount of Ras-GTP in HASMC (157% ± 37 S.E.M.), confirming that also in these cells LTD_4 _is able to activate the small G protein Ras at a concentration relevant for its pathophysiological role in HASMC proliferation (Fig. 6E).

### Effect of wortmannin and LY294002 on LTD_4_-induced DNA synthesis, EGF-R and ERK1/2 phosphorylation

**Figure 7:**
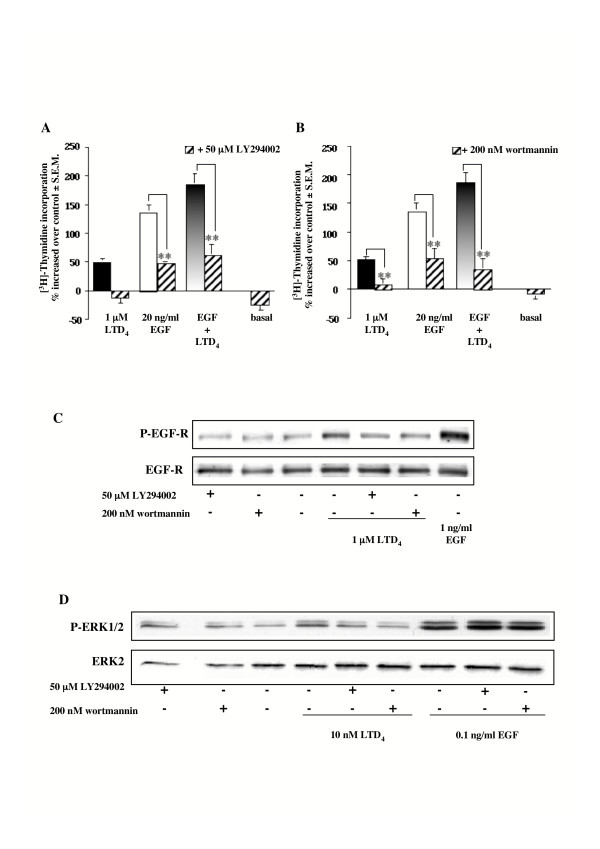
**Effect of LY294002 and wortmannin on [^3^H]thymidine incorporation, EGF-R and ERK1/2 phosphorylation induced by LTD_4 _or EGF**. (A-B) Increase of [^3^H]thymidine incorporation induced by 1 μM LTD_4 _and 20 ng/ml EGF alone and in combination in the absence and presence of (A) 50 μM LY294002 or (B) 200 nM wortmannin (30 minutes pretreatment). Control is represented by MEM additioned with 1% FBS. The results are presented as mean ± S.E.M. of at least three experiments performed in triplicate on at least two different cell lines. **P < 0.01 (one-way ANOVA). (C) EGF-R phosphorylation induced by 1 μM LTD_4 _(3 minutes), in the absence and presence 50 μM LY294002 or 200 nM wortmannin (30 minutes pretreatment). 1 ng/ml EGF was used as an internal control. (D) ERK1/2 phosphorylation induced by 10 nM LTD_4 _and 0.1 ng/ml EGF (5 minutes) in the absence and presence of 50 μM LY294002 or 200 nM wortmannin (30 minutes pretreatment). The results presented are representative of at least three experiments performed on two different cell lines.

It has been previously suggested that different isoforms of PI3K might be involved in the mitogenic signal induced by G_i_-coupled receptors. Thus, we tested two different PI3K inhibitors, i.e. wortmannin and LY29004, and found that 30 minutes pretreatment blunted LTD_4_-induced DNA synthesis. On the contrary, both compounds were only partially able to inhibit [^3^H]thymidine incorporation produced by EGF alone or in combination with LTD_4 _(Fig. 7A–B). Surprisingly, a partial inhibition (~65% for both compounds) was observed on EGF-R transactivation (Fig. 7C). Furthermore, both inhibitors totally ablated LTD_4_-induced ERK1/2 phosphorylation without having an effect on EGF-stimulated MAPK activation (Fig. 7D).

### LTD_4_-induced increase in ROS, and effect of NAC on EGF-R transactivation and MAPK activation

**Figure 8:**
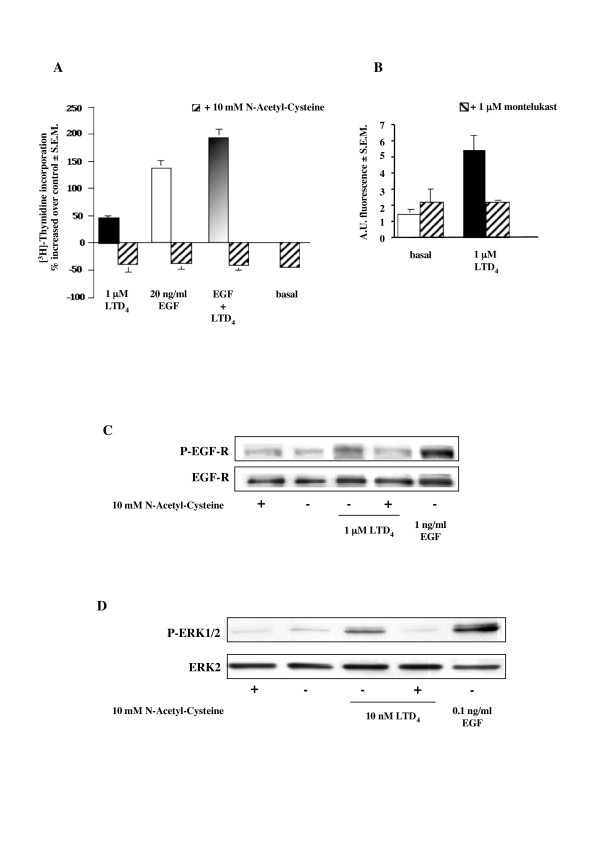
**LTD_4_-induced ROS generation and effect of NAC on [^3^H]thymidine incorporation, EGF-R and ERK1/2 phosphorylation**. (A) Increase of [^3^H]thymidine incorporation induced by 1 μM LTD_4 _and 20 ng/ml EGF alone and in combination in the absence and presence of 10 mM NAC (1 h pretreatment). Control is represented by MEM additioned with 1% FBS. The results are presented as mean ± S.E.M. of four experiments performed in triplicate on two different cell lines. (B) Increase in ROS generation induced by 1 μM LTD_4 _(5 min) in the absence and presence of 1 μM of the CysLT_1_-R antagonist montelukast (30 min pretreatment). Control is represented by MEM additioned with 1% FBS. The results are presented as mean ± S.E.M. of four experiments performed in triplicate on two different cell lines. (C) EGF-R phosphorylation induced by 1 μM LTD_4 _(3 minutes), in the absence and presence of 10 mM NAC (1 h pretreatment). 1 ng/ml EGF was used as an internal control. (D) ERK1/2 phosphorylation induced by 10 nM LTD_4 _and 0.1 ng/ml EGF (5 minutes) in the absence and presence of 10 mM NAC (1 h pretreatment). The results presented are representative of at least three experiments performed on two different cell lines.

Production of ROS has been noted upon growth factor stimulation of arterial smooth muscle cells [43] and bovine tracheal myocytes [44]. We show here that pretreatment for 1 h with 10 mM of the antioxidant NAC, a ROS scavenger, completely inhibits LTD_4_-induced [^3^H]thymidine incorporation (Fig. 8A). Similarly, NAC was also able to entirely inhibit EGF-induced DNA synthesis (Fig. 8A). Thus, to test whether LTD_4 _treatment induces the intracellular generation of ROS, HASMC were loaded with DCFH-DA (10 μM), and stimulated with either LTD_4 _or PDGF (as internal control).

LTD_4 _at the concentration of 1 μM was observed to induce almost a 4-fold increase of fluorescence over baseline, within 5 min (AU = 5.50 ± 1.0 S.E.M vs. AU = 1.42 ± 0.21 S.E.M, P < 0.01). This effect was specifically inhibited by a 30 min pretreatment with 1 μM of the specific CysLT_1_-R antagonist montelukast (Fig. 8B). Finally, we also show that NAC completely ablated LTD_4_-induced EGF-R phosphorylation (Fig. 8C) and MAPK activation (Fig. 8D) as well as EGF-R autophosphorylation (data not shown).

## Discussion

Classical view of molecular pharmacology has always ascribed activation of proliferation to RTKs, while GPCRs have been considered involved in the activation or inhibition of enzymes and ion channels to control second messenger intracellular levels and to activate second messenger-regulated serine/threonine kinases. However, pathways for activation of nuclear transcription in response to GPCR stimulation have now been clearly delineated and involve an always-increasing number of receptors [25].

It has been previously shown that in HASMC LTD_4 _is able to increase DNA synthesis [15], particularly in combination with EGF [14], without investigating the mechanism(s) through which this occurs. We demonstrate here for the first time that LTD_4 _proliferative effect requires EGF-R phosphorylation through the activation of a CysLT_1_-R, PI3K, and ROS production. We also demonstrate that, both EGF-R transactivation and DNA synthesis are strongly dependent upon a G_i/o _protein in these cells. Finally, these LTD_4_-mediated effects were found to induce, downstream of the EGF-R, the activation of the Src-Ras-ERK1/2 pathway.

Functional data obtained with thymidine incorporation confirmed that LTD_4 _is able to potentiate EGF-induced mitogenesis [14]. However, at variance with this report, LTD_4 _was found to increase thymidine incorporation by itself, as already suggested by others [15]. In addition, these effects appear almost completely sensitive to the receptor antagonists zafirlukast and pranlukast, indicative of a pharmacological profile of a classical CysLT_1_-R, again in agreement with previously published data [15]. Thus, our findings seems to corroborate other two reports, in which another specific CysLT_1_-R antagonist, montelukast, was found to attenuate airway remodeling in animal model of asthma [18,45]. However, as CysLT_2_-R is expressed in HASMC [35], we cannot completely rule out the hypothesis of its partial involvement but since the pharmacological profile has been confirmed also at two other end-points (i.e. EGF-R phosphorylation and MAPK activation), we speculate that the CysLT_2_-R is not, or at most marginally, involved in the LTD_4_-mediated effects described here.

Several lines of evidence suggest that recruitment of the EGF-R tyrosine kinase is an essential step for the mitogenic stimulus induced by a number of GPCRs [27] and, sometimes, their effect has been found to be additive or synergistic to RTK-mediated growth. However, it is clear that the signaling events that mediate such augmented growth vary depending on cell line or tissue and remain to be fully characterized in many natural systems, particularly in HASMC [12]. We report here that in HASMC LTD_4_-induced DNA synthesis appears to be totally dependent on EGF-R tyrosine kinase activity. Indeed, LTD_4 _is able to transactivate EGF-R in a time and dose-dependent manner, and to potentiate EGF-stimulated autophosphorylation, in agreement with the additive mechanism of potentiation of EGF-induced thymidine incorporation. These data are consistent with previous observation that cysteinyl-LTs mediate part of ovalbumin-induced lung effects in mice via EGF-R transactivation [46]. Furthermore, this effect can be predominantly ascribed to a CysLT_1_-R. As mentioned before, synergy between RTKs and GPCRs has been already reported in HASMC, despite numerous inflammatory or contractile agents, including thrombin, histamine, and carbachol were not found to cause EGF-R tyrosine phosphorylation, nor did they increase EGF-stimulated autophosphorylation [30].

Interestingly, PTX pretreatment totally inhibited LTD_4_-induced thymidine incorporation as well as LTD_4_-induced EGF-R transactivation, demonstrating the involvement of a G_i/o _protein. Thus, CysLT_1_-R, classically known to be a G_q/11_-coupled receptor in many systems [21], is also coupled to a G_i/o _protein in HASMC. These data are in good agreement with our previous observations that LTD_4 _is only marginally able to induce a cytosolic Ca^2+ ^transient in these cells [33], and that CysLT_1_-R is simultaneously coupled to G_q/11 _and G_i/o _proteins (promiscuous coupling) in another natural expressing systems such as the human monocyte/macrophage-like U937 cells [22].

CysLT_1_-induced EGF-R transactivation in HASMC did not seem to involve Src, as demonstrated by the inability of two different Src kinase inhibitors to significantly affect LTD_4_-induced EGF-R phosphorylation. We also excluded that EGF-R transactivation might require metalloproteinase cleavage of proHB-EGF as well as PKC activity [47]. In fact, neither the HB-EGF inhibitor CRM197, nor GF109203X, a specific PKC inhibitor, affected LTD_4_-induced EGF-R phosphorylation (data not shown). Conversely, EGF-R transactivation seems to require the involvement of a PI3K, likely activated by the βγ complex of G_i_, which, in turn, is known to activate a number of downstream signaling molecules, including different PTKs [48].

However, LTD_4_-stimulated DNA synthesis was sensitive to Src inhibition, which suggests the involvement of Src kinase downstream of EGF-R phosphorylation [49], and, in addition, the activation of Ras and ERK1/2. Indeed, a specific MEK1 inhibitor fully prevented LTD_4_-induced DNA synthesis, and inhibited the potentiating effect of LTD_4 _on EGF-stimulated thymidine incorporation, indicative of the involvement of the MAPK cascade. These data have been confirmed by demonstrating that LTD_4 _was able to increase ERK1/2 phosphorylation and to augment EGF-induced MAPK activation, again with a pharmacological profile characteristic of a CysLT_1_-R, and that both effects were sensitive to Src inhibition. Furthermore, LTD_4 _was observed to increase the amount of active Ras (Ras-GTP), a capacity that we already demonstrated in U937 cells [26].

Consistent with a predominant role of EGF-R transactivation, AG1478 and PTX strongly reduced LTD_4_-stimulated ERK1/2 phosphorylation. Furthermore, while wortmannin and LY294002 had only a negligible effect on EGF-stimulated MAPK activation, they produced a significant inhibition of LTD_4_-induced ERK1/2 phosphorylation. These data, therefore, confirm that PI3K activation is an early event upstream of EGF-R phosphorylation in the signaling cascade linking CysLT_1_-R to thymidine incorporation. Usually, the mitogenic effect caused by EGF involves the activation of a PI3K, but this pathway is parallel to and independent from ERK1/2 phosphorylation. Accordingly, the MEK1 inhibitor was only partially able to inhibit EGF-induced thymidine incorporation. These observations are in agreement with the paradigm that G_i_-coupled receptor- and Gβγ-stimulated MAPK activation is attenuated by inhibition of PI3K acting at a point upstream of Ras, and that this pathway requires a tyrosine kinase and Raf [25].

Looking for a mechanism linking CysLT_1_-R activation and EGF-R phosphorylation, intriguingly we observed that LTD_4 _rapidly increased ROS formation in HASMC, an effect specifically inhibited by the antagonist montelukast. Indeed, different GPCRs have been suggested to increase ROS, which, in turn, inactivate PTP that negatively control RTK activity [50], and therefore, lead to RTK transactivation [51]. While it seems clear that ROS play a role in ASMC mitogenesis, the relevant downstream effectors are not precisely known [29,52]. In this respect, very recently it has been suggested that activation of PI3K may increase ROS formation [53]. In agreement with our results, LTD_4 _has been proposed to increase production of superoxide anion [54], while, more recently, zafirlukast has been shown to interfere with the release of ROS during respiratory bursts of human polymorphonuclear neutrophils or eosinophils [55,56]. Accordingly, we observed that the ROS scavenger NAC inhibited LTD_4_-induced EGF-R transactivation, MAPK activation and thymidine incorporation.

## Conclusion

In conclusion, our data demonstrate that LTD_4_, the most potent bronchoconstrictor yet identified, might potentiate EGF-induced mitogenesis in HASMC, activating a CysLT_1_-R coupled to a G_i _protein to cause EGF-R transactivation through the intervention of PI3K and ROS. This EGF-R induced activation triggers, downstream, the classical Src-Ras-ERK1/2 pathway to control G1 progression and cell proliferation. Furthermore, because interaction between GPCR and RTK might be exceedingly complex and potentially occurs at multiple levels, we may not completely rule out the hypothesis that a second cooperative G_q/11 _pathway might contribute to the LTD_4_-induced effects in HASMC [57]. This possibility is the subject of ongoing investigations in our laboratory.

Taken together, these data corroborate a second, and potentially more important, role for cysteinyl-LTs in modulating cell physiology, from epithelial [40,58] to mesangial [28] or smooth muscle cells [15]. Thus, these lipid mediators produced in large quantities by the inflammatory cells infiltrating hyperresponsive airway walls, an hallmark of chronic airways disorders such as asthma and chronic obstructive pulmonary disease, might significantly contribute to smooth muscle cell hyperplasia associated with these diseases either directly, or, mainly, potentiating growth factor-induced cell proliferation. Hence, long-term inhibition of airway remodeling might disclose new and yet underestimated effects for LT modifiers in the chronic therapy of asthma. Considering the effect of the ROS scavenger also on EGF-induced proliferation, here we propose that an increase in ROS might be a key component not only of the cysteinyl-LTs enhanced proliferative response, but more generally of the airway remodeling associated with chronic asthma.

## Competing interests

The author(s) declare that they have no competing interests.

## Authors' contributions

SR helped in study design and data analysis, was involved in cell culture and carried out RT-PCR, binding, *in vitro *cell proliferation as well MAPK phosphorylation studies.

SC was involved in cell culture and performed *in vitro *cell proliferation, EGF-R and MAPK phosphorylation studies.

BV was the expert for ROS and was involved in the *in vitro *studies on ROS generation and subsequent data analysis.

VC conceived and designed the study and participated to the manuscript preparation.

GER participated in the design of the experiments, coordination and manuscript preparation.
